# Barriers to Autism Spectrum Disorder Diagnosis for Young Women and Girls: a Systematic Review

**DOI:** 10.1007/s40489-020-00225-8

**Published:** 2020-10-29

**Authors:** Georgia Lockwood Estrin, Victoria Milner, Debbie Spain, Francesca Happé, Emma Colvert

**Affiliations:** 1 Centre for Brain and Cognitive Development, Birkbeck, University of London, Malet Street, WC1E 7HX London, UK; 2 Social, Genetic and Developmental Psychiatry Centre, Institute of Psychiatry, Psychology and Neuroscience, King’s College London, Denmark Hill, SE5 8AF London, UK

**Keywords:** Autism, Diagnosis, Female, Gender differences, Barriers

## Abstract

There is increased recognition that women and girls with autism spectrum disorders (ASD) are underserved by the clinical criteria and processes required to receive a diagnosis. This mixed-methods systematic review aimed to identify key barriers to obtaining an ASD diagnosis in girls and young women under 21 years. Six themes were identified that focused on perceived gendered symptoms, namely behavioural problems, social and communication abilities, language, relationships, additional diagnoses/difficulties and restricted and repetitive behaviours and interests. Five themes were identified as (parental) perceived barriers to diagnosis, namely compensatory behaviours, parental concerns, others’ perceptions, lack of information/resources and clinician bias. This review highlights the importance of enhancing widespread understanding and recognition of ASD presentation in females across development. PROSPERO Centre for Reviews and Dissemination (ID 2018 CRD42018087235)

**Supplementary Information:**

The online version of this article (10.1007/s40489-020-00225-8) contains supplementary material, which is available to authorized users.

Autism spectrum disorder (ASD) is characterized by social and communication difficulties, as well as restricted interests and repetitive behaviours. Historically, ASD has been thought of as a predominantly male disorder, with a recent review and meta-analysis suggesting a male:female ratio of 3:1 (Loomes et al. 2017). While the high male to female ratio likely reflects aspects of the aetiology of ASD, it is also conceivable that there are biases in perception, assessment, and/or diagnosis of ASD for females. In line with this latter suggestion, in population-based samples and with thorough ascertainment, the gender ratio can fall to as low as 1.8:1 (Mattila et al. 2011).

Some autistic females may present similarly to autistic males. However, there is increasing evidence that a female autism phenotype may exist. Distinguishing between male and female phenotypes of ASD is a complex endeavour. This is partly due to methodological bias, resulting from the inclusion of predominantly male samples, as well as the use of clinical tools designed to fit the male ASD phenotype. Such tools may not be sensitive to the differing distribution of autistic traits between males and females (Young et al. 2018). This, in addition to possible differences in the female ASD phenotype (see Rubenstein et al. 2015 for a review), indicates that females may be poorly served by the current conceptualisation and recognition of ASD. There are increasing concerns that autistic females are being missed, diagnosed later than males or misdiagnosed (Giarelli et al. 2010; Holtmann et al. 2007; Loomes et al. 2017; Shattuck et al. 2009).

In addition to potential sex^1^ differences in clinical presentation (Lai et al. 2014; May et al. 2014; Young et al. 2018), it is increasingly recognised that autistic people can hide their ASD symptoms or use compensatory behaviours to mitigate social challenges (Gould and Ashton-Smith 2011; Hull et al. 2017; Livingston et al. 2019; Tierney et al. 2016). This has been reported to be more common in females than males and associated with different neural processes (Lai et al. 2019). If diagnostically relevant behaviours are masked, this can exacerbate the possibility of delayed or missed diagnosis.

Another key reason for misdiagnosis may be the pervasive perception that ASD is a ‘boy’s disorder’ (Riley-Hall 2012, p. 37). This is supported by evidence that boys are referred for a diagnostic assessment 10 times more often than girls (Wilkinson 2008). A clear delay in diagnosis for cognitively able girls compared to boys is also evident, despite there being no difference in the number of appointments with healthcare professionals during the diagnostic process (Siklos and Kerns 2007), the age at which parents express concern (Begeer et al. 2013), or the duration of assessment (Wilson et al. 2016). It has also been shown that even with comparable levels of symptom severity, females are less likely than males to receive a diagnosis (Geelhand et al. 2019; Russell et al. 2011).

Despite an increased research effort to characterise the female presentation of ASD and barriers to diagnosis, there has been no systematic review synthesising the results of these studies to date. The current paper reports a mixed-methods systematic review aiming to identify key barriers to obtaining an ASD diagnosis in females under 21 years old. This age group was focused upon as previous research has suggested that barriers to diagnosis differ in adult women with ASD compared to girls and young women under 21 years (Rutherford et al. 2016). This review addresses the following research questions:

1. Do sex differences in ASD behaviours constitute barriers to ASD diagnosis in girls and young women?
2. What are the barriers to ASD diagnosis in girls and young women from the perspectives of (a) autistic individuals, (b) their parents and family members, (c) teachers, and (d) health professionals?

## Methods

The review protocol was developed a priori and registered at PROSPERO Centre for Reviews and Dissemination (ID 2018 CRD42018087235).

### Search Strategy

Four electronic databases (PsycInfo, EMBASE, Medline, CINAHL) were searched to include articles published up until October 2018. Subject headings, keywords, and MeSH terms were related to the following terms:

Autism spectrum disorders (including Autism, Asperger) AND barriers (including barrier, challenge, access) AND diagnosis (including identification, detect) AND gender (including male/female, girl/boy, daughter) AND children/young people (including toddler, child, adolescent, youth). For objective 1: AND symptoms/behaviours (including camouflage, behaviour, symptom, mask, mimic), for objective 2: AND perception (including understanding, experience) AND health worker (including clinician, nurse, doctor) OR teacher OR parent/family (including mum/mother, dad/father). The search strategy was developed in EMBASE and subsequently adapted for each database (see Appendix 1 for full search strategy). These searches were limited to studies published in English.

Citation and reference searches were carried out for all relevant papers identified through the database searches. Authors of relevant papers and experts in the field were contacted for recommendations regarding other potential publications to consider for inclusion.

The first screening of search results considered studies’ titles, abstracts, and keywords. Second, full-text reports were then obtained for all potentially relevant studies and screened against the full inclusion criteria. For both the first screening stage and the second full-text assessment for eligibility, three researchers (GLE, VM, and EC) separately assessed an equal proportion of the search results, and 10% of these were compared for consistency. Discrepancies were resolved through discussion. Study authors were contacted for clarification, where necessary.

The inclusion criteria for the review were peer-reviewed English language publications reporting primary qualitative and/or quantitative studies. The studies reported on issues related to barriers or challenges to ASD diagnosis in girls and young women and for objective 1, recruited samples of girls or young women (aged 21 or younger) with a diagnosis of ASD or measured ASD traits. For objective 2, in terms of informants, qualitative data could be reported by girls or young women with ASD, family members, healthcare providers, or teachers. Papers were excluded if they solely focused on gendered ASD symptoms/behaviours, without addressing barriers to ASD diagnosis; described differences in presentation or diagnosis by gender group, but did not explore factors accounting for these differences; did not disaggregate data by gender; or investigated developmental disorders without disaggregation by type of neurodevelopmental disorder (e.g. cases of Asperger’s vs Down’s syndrome).

### Data Extraction, Analysis, and Synthesis

Data were extracted on (1) publication details (including date, authors, title), (2) study design (country, setting, design, sample size; recruitment, eligibility for participation), (3) participant information (age, gender, diagnosis, comorbidities, socio-demographics, family characteristics), (4) methodology (qualitative/quantitative data collection, measurement of ASD symptoms/diagnosis, and covariables), (5) analysis methods, (6) results and conclusions and (7) study limitations. Experimental data were extracted from participants’ quotes, statistical results, and from authors’ interpretations and discussions regarding barriers to diagnosis of ASD.

Three researchers (GLE, VM, and EC) conducted an equal proportion of data extraction and assessment of methodological quality for the included studies, and 10% of data extraction forms were compared for consistency. Methodological quality of the included studies was assessed using the qualitative Critical Appraisal Skills Programme (CASP 2018) checklist, or an adapted CASP checklist (see Appendix 2) for quantitative research studies. Papers were deemed to be of ‘high’, ‘moderate’, or ‘low’ quality based on previously cited cut-off scores for qualitative papers (high, 9–10; moderate, 7.5–9; low, 6–7.5) (Butler et al. 2016) and adapted for quantitative papers (high, 6.25–7; moderate, 5.25–6; low, 4–5); scores below the ‘low’ category were excluded from the synthesis. The adapted quality appraisal was piloted on 10% of the included papers during data extraction.

Data from quantitative and qualitative studies were synthesised in a narrative review to summarize, explain, and interpret evidence from the included papers; thematic analysis was conducted to draw out themes from the data, as outlined by Mays et al. (2005) on conducting systematic reviews from mixed evidence sources. Following thematic analysis guidelines (Braun and Clarke 2006), three authors (GLE, VM, EC) individually developed codes through examining evidence extracted from included papers. These codes were refined during three sets of discussions between authors (GLE, VM, EC), until agreement was reached. Themes were identified and then refined upon immersion of the data and agreed upon by coders. Themes were checked to ensure consistency and clarity. This process was repeated for symptoms and barriers separately.

## Results

**Fig. 1:**
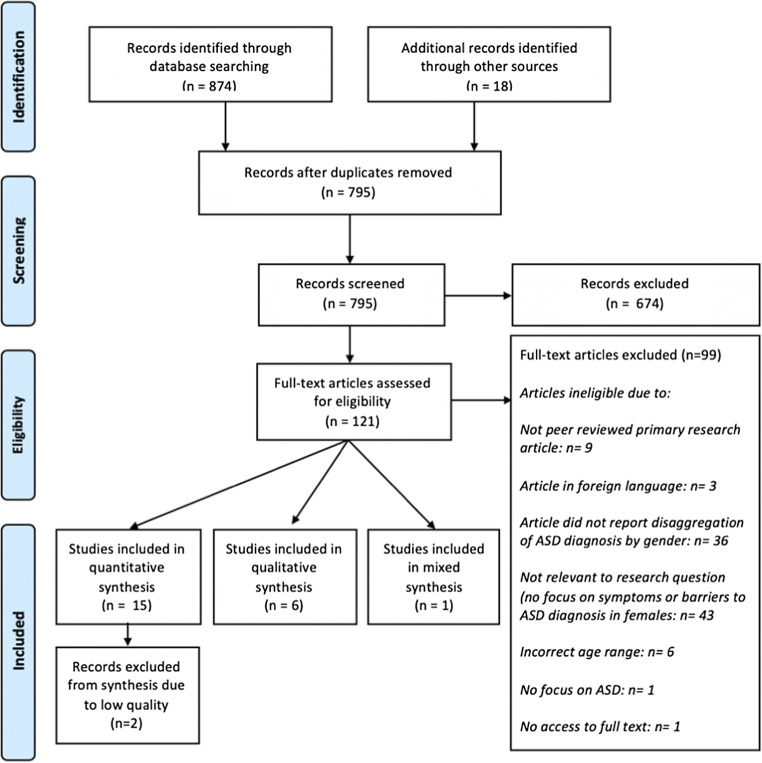
PRISMA flow diagram

An initial sample of 795 papers was found by the database searches and were screened. A total of 13 quantitative, six qualitative, and one mixed-methods papers were included in this review (see Fig. 1, which summarises the numbers and reasons for exclusion at each stage). A total of 23,760 participants were included in this review; this included a total of 3197 females with a diagnosis of ASD, 92 parents/family members of girls/young women diagnosed with ASD, zero teachers, and three professional providers.

### Methodological Quality

For quantitative papers, two out of 15 papers were excluded due to poor quality. Of those included, five papers were scored ‘high’, six papers ‘moderate’, and two papers ‘low’ for quality. For the qualitative papers, three were scored ‘high’, two scored ‘moderate’, and one scored ‘low’ for quality. The one mixed-method paper included was scored ‘moderate’ (Table 1).

**Table 1:** Overview of included papers

	Author	Aim	Design	Sample	Main findings on barriers to ASD diagnosis in females	Paper quality
Qualitative	Beteta	To describe lived experiences of adolescent females with ASD	In-depth semi-structured interviews; Field notes, observations, and artefacts	Clinical sample.- *n* = 4 AS females Age (range), 17–19 years Country, USA	– Females with AS share similar interests with neurotypical peers. – Girls had been victims of bullying and suffered anxiety and frustration.	Low
	Cridland	To investigate the experiences of girls with ASD during adolescence	In-depth semi-structured interviews	Clinical sample *n* = 5 ASD females *n* = 5 mothers Age (range), 12–17 years Country, Australia	– Challenges for obtaining a diagnosis: symptom presentation, imitation of social behaviour, higher incidence of ASD in boys, misdiagnosis, and reluctance from health professionals to provide diagnosis. – Participants discussed experiences of living with a disorder associated with boys. – Issues relevant to girls: difficulties socialising with peers, sex-specific puberty issues, and sexual vulnerabilities.	High
	Watson	To understand the diagnostic process experience	In-depth semi-structured interviews	Clinical sample *n* = 12 ASD adolescent girls *n* = 20 mothers, fathers *n* = 5 siblings *n* = 3 professional providers Age (mean (range)), 14 years (13–17 years) Country, USA	– All girls had at least one co-occurring diagnosis. – Parents perceived autism and AS as a ‘boy’s disorder’ linked to delays in picking up ASD symptoms. – No parents identified a linear pathway for diagnosis for their daughters. – Lack of information from professionals that validated parental concerns. – Factors associated with earlier diagnosis: prominent autism symptoms, higher levels of maternal education, higher socioeconomic status.	High
	Navot	To investigate the maternal experience of raising a daughter with ASD	In-depth semi-structured interviews	Clinical sample *n* = 11 ASD females *n* = 11 mothers Age (mean (SD)), 14.8 (± 3.3) years Country, USA	Themes were identified, including: – scepticism and delayed diagnosis – disbelief from others – lack of information about girls with ASD	Moderate
	Cook	To explore experiences of learning, friendships, and bullying in girls with ASD	In-depth semi-structured interviews	Clinical sample *n* = 11 ASD females *N* = 11 mothers, fathers Age (range), 11–17 years Country, UK	Three core themes identified as follows: – Motivation to have friends – Challenges faced by girls with autism, e.g. social isolation; bullying; and absenteeism, stress, and anxiety – Masking autism	Moderate
	Mademtzi	To examine the educational and therapeutic needs of females with ASD	Focus group discussions	Clinical sample *n* = 40 families with daughter with ASD Age (mean (range)), 15.9 years (4–29 years) Country, USA	– Families experienced difficulty obtaining early diagnosis and problems having to justify requests for services – Challenges relevant to females were sexual vulnerability, same-sex friendships, feminine self-care, and increased barriers to accessing services. – Financial concerns to cover services.	High
Quantitative	Giarelli	To examine sex differences in age at first developmental evaluation, diagnosis, influence of cognitive impairment, and sex-specific behavioural characteristics	Secondary data analysis of a population-based study of the prevalence of ASD	Community sample *n* = 2568 (*n* = 488 ASD females^**^) Age, 8 years Country, USA	– No sex differences in age at first evaluation or diagnosis. – Compared to boys, girls less likely to have a documented diagnosis when adjusted for cognitive impairment status and had more seizure-like behaviour.	High
	Russell	To examine social and demographic factors in receiving a diagnosis of ASD	Retrospective secondary data analysis of a longitudinal cohort study	Community sample *n* = 71 (*n* = ASD females) Age (range), 2.5–4 years Country, UK	– Boys more likely to have severe autistic traits, whether diagnosed with ASD or not. – Girls less likely to be diagnosed than boys, irrespective of symptom severity.	High
	Dworzynski	To explore sex differences in autistic traits in relation to diagnosis	Population-based study	Community sample *n* = 189 (*n* = 29 ASD females^**^) Age (range), 10–12 years. Country, UK	− Girls, but not boys, meeting diagnostic criteria for ASD had significantly more additional problems (low intellectual level, behavioural difficulties) than peers with similarly high CAST scores who did not meet diagnostic criteria.	High
	Begeer	To examine differences by sex in the timing of identification of individuals with ASD	Survey	Community sample *n* = 2272 (*n* = 432 ASD females) Age (range): children, 0–18 years adults, 18–85 years Country, Netherlands	– Girls identified 1.8 years later than boys among children with AS. – No delayed identification for girls with ASD or PDD-NOS.	High
	Head	To examine the female presentation of ASD	Observational, cross-sectional study	Clinical sample *n* = 101 (*n* = 25 ASD females) Age (mean (SD)), 13.56 (±2.10) years Country, Australia	– Females demonstrated higher levels of sociability and friendship compared to males. – Diagnosis was a significant predictor of participants’ levels of sociability and friendship. – Children with ASD demonstrated lower levels of sociability and friendship compared to TD counterparts.	Low
	Rynkiewicz	To examine non-verbal communication using a novel technique	Experimental, cross-sectional study	Clinical sample *n* = 33 (*n* = 16 ASD females) Age (mean (SD)), 8.06 (± 1.57) years Country, Poland	– Girls with ASD used gestures more vividly than boys. – Girls with ASD made significantly more mistakes than boys on an emotion recognition test. – Current communication skills of boys with ASD significantly better than those of girls. – Girls and boys with ASD improved in their social and communication abilities over the lifetime. – Number of stereotypic behaviours in boys significantly decreased over life but remained constant in girls.	Low
	Salomone	To investigate age at diagnosis of ASD between girls and boys	Secondary data analysis from an online survey	Community sample *n* = 1410 (*n* = 257 ASD females) Age, under 7 years Country, multisite European study	− Females with complex phrase speech diagnosed later than males with the same verbal ability level.	Moderate
	Duvekot	To investigate behavioural characteristics and ASD diagnosis in girls and boys	Observational, multiple centre cross-sectional study	Clinical sample *N* = 231 (*n* = 64 ASD females) Age (mean), 7.9 years Country, Netherlands	– Higher scores for RRBI less predictive of an ASD diagnosis in girls than in boys. – Girls more likely to be diagnosed with ASD when they had higher total levels of behavioural problems. − Sensory symptoms equally predictive of an ASD diagnosis in girls as in boys.	Moderate
	Little	To examine caregivers’ primary concerns prior to a diagnostic evaluation	Observational study, clinical comparison of caregiver’s concerns	Community sample Total = 242 (*n* = 63 ASD females) Age (mean), 72.14 months. Country, USA	− Caregivers were more concerned about social interaction in boys than girls with ASD.	Moderate
	Mandy	To investigate the course of ASD development	Longitudinal cohort study	Community sample *n* = 9744 (*n* = 623 ASD females^**^) Age, 7, 10, 13, and 16 years. Country, UK	– At 7 years, males had higher autistic social traits (AST) than females; these declined between 7 and 10 years and then rose from 10 to 16 years. – At 16 years, males had lower ASTs than at 7 years, females had higher ASTs than at 7 years. − At 16 years females did not have lower ASTs than males applying across the IQ range.	High
	Petrou	To investigate age at ASD diagnosis between girls and boys.	Secondary data analysis from UK database	Clinical sample, two groups Diagnosed at < 60 months *n* = 1873 (*n* = 324 ASD females) Diagnosed at ≥ 60 months *n* = 1462 (*n* = 264 ASD females) Age (mean (SD)) Diagnosed at < 60 months, 78.2 (± 40.5) months Diagnosed at ≥ 60 months, 133.6 (± 38.8) months Country, UK	– ≥ 60 months of age for diagnosis, girls diagnosed later than boys (on average by 12 months), no difference found under 60 months of age. – Frequency and number of co-existing conditions were associated with age at diagnosis for girls and boys. – In boys, not girls, type of ASD diagnosis, language level, additional diagnoses, and frequency of co-existing conditions were associated with age at diagnosis.	Moderate
	Ramsey	To examine the impact of sex and diagnosis	Observational, cross-sectional study	Community sample *n* = 669 (*n* = 74 ASD females) Age (range), 25.9 (± 5.0) years Country, USA	– No sex differences found in parents’ concerns for ASD toddlers; in at-risk sample, parents of boys endorsed more ASD symptoms. – Parents expressed RRBI concerns for boys 1.74 times more often than for girls. – Parents of boys were 2.43 times more likely to name ASD as a concern than parents of girls.	Moderate
	Tillmann	To investigate sex differences in ASD symptomatology	Secondary data analysis across multiple cross-sectional studies	Community sample *n* = 2684 (*n* = 464 ASD females) Age (mean (SD)), 11.2 (± 9.5) years Country, multisite European study	– Rates of early childhood RRBI lower in females than males. – Comparable levels of social interaction and communication difficulties in females and males. – No sex differences for ASD severity.	Moderate
Mixed	Dean	To investigate social behaviours in boys and girls and its role in masking ASD symptoms.	Secondary data analysis from an RCT	Clinical sample *n* = 73 (*n* = 24 ASD females) Age (mean (SD)), 7.75 (± 1.22) years Country, USA	− Girls spent more time on joint engagement activities than boys with and without ASD.	Moderate

^**^ASD diagnosis defined by high/positive scores on ASD screening questionnaires such as SCDC, DAWBA, or clinical recommendation, rather than a clinical diagnosis

*AS*, Asperger syndrome; *PDD-NOS*, pervasive developmental disorder–not otherwise specified; *CAS*T, Childhood Autism Spectrum Test; *TD*, typically developing; *RRBI*: restrictive and repetitive behaviours and interests

N.B. ‘Age’ denotes age of participants with an ASD diagnosis

**Table 2:** Gendered symptoms as a barrier to diagnosis

Gendered symptoms	Relevant papers	Identified symptom/behaviour	Main findings relevant to gendered symptoms
Behavioural problems	Giarelli	1. Aggression 2. Hyperactivity or attention deficits 3. Seizure-like activity	Boys without a documented ASD classification had more externalizing behaviours than girls, such as the following: 1. Aggression (X2 (df = 1, *n* = 1071) = 7.36, *p* = .004). 2. Hyperactivity or attention deficits (X2 (df = 1, *n* = 1071) = 8.38, *p* = .003]). 3. Girls without an ASD classification were more likely than boys to have staring spells and seizure-like activity (X2 (df = 1, *n* = 1071) = 12.38, *p* = .001).
	Dworzynski	1. Hyperactivity 2. Behavioural difficulties, within genders 3. Behaviour difficulties, girls	Within the gender groups, diagnosed versus high-CAST girls had significantly higher reported levels of; 1. Hyperactivity (*t*(51) = 3.29, *p* = .002, partial *n*^2^ = 0.18). 2. Higher overall behavioural problem scores (*t*(51) = 3.08, *p* = .003, partial *n*^2^ = 0.16). 3. Odds 5.4 times higher for diagnosed versus high-CAST girls, to show high levels of behavioural difficulties.
	Duvekot	1. Behavioural difficulties between genders	1. A significant interaction between gender and the total CBCL score: girls were more likely to be diagnosed with ASD when they had higher total levels of behavioural problems (OR = 2.40, 95% CI 1.13–5.29), whereas this effect was not present in boys (OR = 0.98, 95% CI = 0.70–1.38).
	Petrou	1. Toileting problems & temper problems 2. Eating problems 3. Co-existing conditions 4. Other	1. Boys and girls were diagnosed earlier if they had a toileting problem (23.7% boys, 20.9% girls) and temper problems (50.6% boys, 49.2% girls). 2. A total of 52.2% of boys and 49.4% of girls had eating problems; boys with an eating problem were diagnosed earlier (β = − 0.019) than boys without, whereas eating problems did not explain any significant variance in age at diagnosis in girls (β = − 0.027). 3. Frequency and number of co-existing conditions was significant for boys (*F*(10,999) = 5.45, *p* < .001, *R*^2^ = 0.052) and for girls (*F*(10, 207) = 2.97, *p* = .002, *R*^2^ = 0.126) and explained a further 1.8% and 9.2%of the variance, respectively. 4. Hyperactivity (47.9% boys, 45.5% girls), sensory problems (55.1% boys, 60.2% girls), and the total number of co-existing conditions did not explain any significant additional variance for either boys or girls.
Language	Dworzynski	1. Language for girls 2. Language for boys	1. OR 4.2 times higher for diagnosed girls to fall 1.5 SDs below the mean verbal IQ compared with their high-CAST counterparts. 2. OR 2.7 times higher for diagnosed boys to fall more than 1.5 SDs below mean verbal IQ compared with high-CAST boys.
	Salomone	1. Language	1. In children with complex phrase speech, age at diagnosis was higher for females (M = 48.57, SD = 1.18) than males (M = 45.69, SD = 0.55), *F*(1, 1245) = 4.869, *p* = .028. No gender effect for non-verbal (*F*(1, 1245) = 2.294, *p* = .130) or minimally verbal children (*F*(1, 1245) = 0.344, *p* = .558). A significant interaction between verbal ability and gender on age at diagnosis, *F*(2, 1245) = 3.519, *p* = 0.030; the effect size was small (partial η^2^ = 0.01).
	Petrou	1. Language for girl 2. Language for boys	1. Language level not significant for girls (*F*(3, 214) = 0.63, *p* = .594, *R*^2^ = 0.009) and explained 0% of variance. 2. Language level significant for boys (*F*(3, 1006) = 6.23, *p* < .001, *R*^2^ = 0.018) and explained a further 0.5% of the variance. Boys whose language repertoire comprised only lower levels of language were diagnosed earlier than boys who spoke in sentences (β = 0.025) but this was not the case for girls (β = − 0.004).
Social communication abilities	Rynkiewicz	1. Emotion recognition 2. Social and communication skills	1. Girls with autism made significantly more mistakes than boys with autism on the Faces Test (emotion recognition). 2. Current communication skills of boys with autism reported by parents in SCQ were significantly better than those of girls with autism. However, both girls with autism and boys with autism improved in the social and communication abilities over the lifetime.
	Mandy	1. Social and communication skills	1. At 7 years, males had higher levels of autism social traits (ASTs) than females (mean raw score difference = 0.88, 95% CI [.72, 1.04]) and were more likely (OR = 1.99; 95% CI 1.82, 2.16) to score in the clinical range on the SCDC. By 16 years, this gender difference had disappeared: males and females had, on average, similar levels of ASTs (mean difference = 0.00, 95% CI [0.19, 0.19]) and were equally likely to score in the SCDC’s clinical range (OR = 0.91, 95% CI, 0.73, 1.10). This was the result of an increase in females’ ASTs between 10 and 16 years.
	Tillmann	1. Social and communication skills	1. A non-significant trend towards higher scores in males was found on the ADI-R communication domain (*p* = .074, *d* = .12). No main effect of sex for ADOS CSS total, ADOS social affect, ADOS RRB (Total *N* = 1420, all *p* > .60) and ADI-R current domain social, communication, and RRB scores (Total *N* = 1030, all *p* > .20) were observed.
RRBIs	Rynkiewicz	1. Stereotypic behaviour	1. The number of stereotypic behaviours in boys significantly decreased over life whereas it remained at a comparable level in girls with autism.
	Duvekot	1. RRB 2. Sensory symptoms	1. OR of 0.41 indicates that an increase of one standard deviation on the RBS-R total scale increased the odds of an ASD diagnosis in girls (OR = 1.10, 95% CI = 0.48–2.45) less than half of what it increased the odds in boys (OR = 2.67, 95% CI = 1.50–4.75). 2. Parent-reported and teacher reported autistic symptoms and parent-reported sensory symptoms significantly predicted an ASD diagnosis irrespective of gender.
	Tillmann	1. RRB	1. Fewer early childhood RRB on the ADI-R in females compared to males. Non-verbal intellectual functioning may attenuate some of the sex differences found in RRB in ASD. 2. For ADOS CSS RRB, there was a significant sex by age interaction (b = −.02, *p* = .004), with females but not males showing significantly lower scores with increasing age. When restricting the analysis to individuals aged 25 or less (retaining 97% of the initial sample), the sex by age interaction was not significant (b = −.01, *p* = .22), suggesting these results are likely to be driven by a small number of older adult male participants with high RRB symptoms.
Relationships	Head	1. Friendships	1. Children with ASD of both genders had a similar reduction in FQ scores. However, ASD females show similar FQ scores to TD males. For both parental and child FQ score ratings gender was found to be a significant predictor of participants FQ scores (*F*(1, 101) = 22.66, *p* < .001, η*p*^2^ = 0.20). An independent samples *t* test was conducted to examine the mean FQ scores between females with ASD and males with ASD. A significant difference was found across gender (t(48) = − 3.64, *p* < .05).
Additional diagnoses/difficulties	Giarelli	1. Cognitive impairment	1. Girls with IQ of 70 or less were less likely than boys with IQ of 70 or less to have a documented diagnosis.
	Petrou	1. Additional diagnosis	1. Boys & girls who had an additional diagnosis were diagnosed later than those who did not have an additional diagnosis (variance not significant for girls).

*CBCL*, Child Behaviour Checklist; *TD*: typically developing; SCQ: Social Communication Questionnaire; CAST: Childhood Autism Spectrum Test; *OR*, odds ratio; *CI*, confidence intervals; *ADOS*, Autism Diagnostic Observation Schedule; *CSS*, Calibrated Severity Score; *ADI-R*, Autism Diagnostic Interview–Revised; *FQ*, Friendship Questionnaire; *RRB*, restrictive and repetitive behaviour

### Gendered Symptoms as a Barrier to Diagnosis

**Fig. 2:**
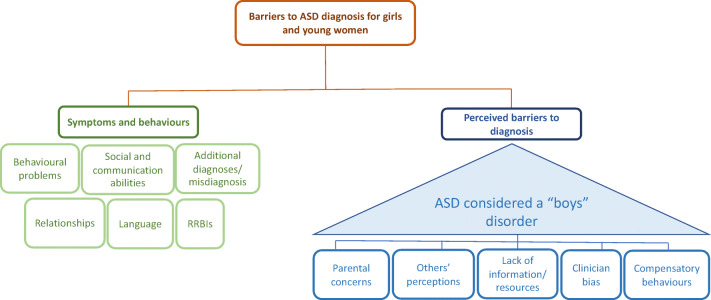
Themes identified

In relation to ASD behaviours constituting barriers to ASD diagnosis in girls and young women, six themes were identified from the literature, i.e. behavioural problems, social and communication abilities, language, relationships, additional diagnoses/difficulties and restricted and repetitive behaviours and interests (RRBIs) (Fig. 2).

### Behavioural Problems

Four papers described comparisons between genders and presented conflicting findings (Duvekot et al. 2017; Dworzynski et al. 2012; Giarelli et al. 2010; Petrou et al. 2018). When comparing females and males with high ASD traits, it was found that males were more likely to have aggression and hyperactivity and/or attention deficits, and females were more likely to exhibit staring and seizure-like activities (Giarelli et al. 2010). Within genders, it was found that ASD girls were more likely to have behavioural difficulties (e.g. hyperactivity), compared to high-trait girls not meeting diagnostic criteria, while boys showed no such difference (Dworzynski et al. 2012). This was also true for low IQ, with girls meeting diagnostic criteria having lower intellectual abilities than those who did not meet the criteria.

Within ASD samples, no gender differences in age of diagnosis were found if toileting or temper problems were present (Petrou et al. 2018), i.e. both males and females being diagnosed earlier if these problems were apparent versus absent. However, boys, but not girls, who had both ASD and eating problems, were diagnosed earlier than those without eating problems. However, Duvekot et al. (2017) found that in a clinic-based sample, having additional behavioural problems increased the likelihood of obtaining an ASD diagnosis in females, whereas this effect was not present for males; this reflects the findings from a high-trait sample (Dworzynski et al. 2012). Arguably, this could be framed as females diagnosed with ASD needing additional behavioural problems to improve their chances of receiving a diagnosis.

### Social Communication Abilities

Three studies described social communication abilities as a possible influencing factor of diagnosis of females with ASD (Mandy et al. 2018; Rynkiewicz et al. 2016; Tillmann et al. 2018).

Mandy et al. (2018) examined the developmental trajectories of autistic social traits (AST) for males and females included in the population-based Avon Longitudinal Study of Parents and Children (ALSPAC), assessed with the parent-report Social Communication Disorders Checklist. Significant differences emerged for the developmental course of AST between genders in this general population sample; at earlier ages (7 years), males showed higher levels of AST. Yet, by early to mid-adolescence (13, 16 years), the gender gap narrowed, irrespective of IQ. It would seem that social difficulties became more pronounced in girls over time, or that compensatory mechanisms such as camouflaging are less successful, against the higher social demands of teenagers.

Rynkiewicz et al.’s (2016) study included the use of the Faces Test to assess emotion recognition in a clinical sample of 5- to 10-year olds and found that girls with ASD made significantly more mistakes than boys, indicating poorer emotion recognition skills. It should be noted that the sample size for this study was small (*n* = 16 females), and all participants were of average or above average IQ and verbally fluent—factors that may limit the generalisability of this finding. The study also included the parent-completed Social Communication Questionnaire (SCQ), on which current communication skills were reported to be significantly better for ASD boys than girls. This again indicates greater problems for diagnosed girls and the possibility of girls needing more ASD traits than boys to gain an ASD diagnosis.

Tillmann et al. (2018) pooled datasets from across European clinical and research sites (*n* = 18 sites across nine countries), thereby providing one of the largest sample sizes to date, to assess the impact of sex and age in terms of scores on the Autism Diagnostic Observation Schedule (ADOS-G, Lord et al. 1989) and Autism Diagnostic Interview (ADI-R Rutter et al. 2003). This study found no significant sex differences relating to current social communication symptoms for either the ADOS or the ADI-R, indicating a similar phenotype in terms of social communication deficits for males and females meeting ASD diagnostic criteria, irrespective of age, in a clinical sample.

### Language

Three studies addressed the issue of language and the role that this might play in terms of diagnosis for males and females (Dworzynski et al. 2012; Petrou et al. 2018; Salomone et al. 2016). Dworzynski et al. (2012) found that in a population-based sample, girls, but not boys, meeting diagnostic criteria for ASD showed significantly lower levels of verbal cognitive ability than peers with similarly high CAST (Childhood Autism Spectrum Test) scores who did not meet criteria for ASD. Salomone et al.’s (2016) finding reflects that of Dworzynski et al.’s (2012) in a lower age group (< 7 years compared with 10–12 years old). Salomone et al. (2016) found that for verbally able individuals (i.e. those with phrase speech) in a population-based sample, girls had a significantly higher age of diagnosis than boys. This gender difference was not seen for non-verbal or minimally verbal children. These results indicate that verbally able girls may be waiting longer for a diagnosis, thereby supporting Dworzynski et al.’s (2012) suggestion that additional language (or other) difficulties are often necessary for girls to receive an ASD diagnosis in contrast to boys. However, in the final paper to examine language skills in relation to diagnosis, Petrou et al. (2018) found that in a clinic-based sample, boys, but not girls, with lower language levels were diagnosed earlier.

### Relationships

Only one study specifically addressed relationships in females compared to males with ASD under 21 years of age (Head et al. 2014). The Australian study with 25 male and 25 female adolescents with high-functioning ASD demonstrated that females with ASD showed similar social skills on a (self-report/parent-report) questionnaire of friendship and social function as typically developing males. The authors go on to identify that if diagnostic criteria in the social communication domain considered these striking gender differences, a larger number of females may go on to be diagnosed with ASD. This is an important finding, however, study limitations should not be ignored; the friendship questionnaire (FQ, Baron-Cohen and Wheelwright 2003) was modified from its original form for adults, and this had not been validated and had relatively low reliability. However, the study also reported that adolescents with ASD scored lower on the FQ compared to neurotypical adolescents, which is strongly supported by the literature, and study findings were consistent when using the FQ reported by adolescents compared to parent-report, providing further validation of this study’s findings.

### RRBIs and Sensory Sensitivities

Three papers reported gender differences in RRBIs relevant to diagnostic barriers (Duvekot et al. 2017; Rynkiewicz et al. 2016; Tillmann et al. 2018). Generally, studies indicated that males show more RRBIs than females (Duvekot et al. 2017; Tillmann et al. 2018), and that RRBIs are more predictive of an ASD diagnosis in males compared to females.

Interestingly, Rynkiewicz et al.’s (2016) cross-sectional study found that the number of stereotyped behaviours in boys significantly decreased between five and 10 years of age, whereas it remained at a consistent level across these ages in girls with ASD. Tillmann et al. (2018) found that girls with an ASD diagnosis exhibited fewer RRBIs than boys, however non-verbal intellectual functioning accounted for and attenuated these differences.

Only one paper reported on sensory sensitivities between genders in relation to diagnosis (Duvekot et al. 2017). This study found that parent-reported sensory symptoms significantly predicted an ASD diagnosis irrespective of gender.

### Additional Diagnoses/Difficulties

Four papers discussed the impact of additional (co-occurring) diagnoses on ASD diagnosis (Cridland et al. 2014; Giarelli et al. 2010; Petrou et al. 2018; Watson 2014). Watson (2014) found that all participants (*n* = 13 females with a clinical ASD diagnosis) reported having a co-occurring condition (e.g. ADHD), with 10 out of the 13 participants receiving their co-occurring diagnosis prior to ASD. Additionally, Cridland et al. (2014) showed parents reported misdiagnosis as a factor leading to delayed diagnosis in females. Petrou et al. (2018) found that boys who had an additional diagnosis were diagnosed significantly later than boys who did not. Although the model was not significant for girls (girls represented 18% of the sample), a trend was observed, whereby girls with an additional diagnosis were diagnosed later than girls who did not have an additional diagnosis.

It has also been suggested that cognitive impairment increases the likelihood of having a documented ASD diagnosis for boys, but not for girls (Giarelli et al. 2010). Girls with an IQ of 70 or less were less likely than boys with an IQ of 70 or less to have a documented ASD diagnosis. This may suggest that once a cognitive impairment had been identified in a female, it is less likely that an ASD assessment will take place.

### Perceived Barriers to Diagnosis

Five themes were identified from the literature, i.e. compensatory behaviours, parental concerns, others’ perceptions, lack of information/resources and clinician bias (see Fig. 2).

### Compensatory Behaviours

Six papers discussed compensatory behaviours as barriers to diagnosis for females (Beteta 2008; Cook et al. 2018; Cridland et al. 2014; Dean et al. 2017; Rynkiewicz et al. 2016; Watson 2014). These papers discussed how females with ASD might go unnoticed due to their behaviours appearing similar to their neurotypical peers.

Dean et al. (2017) observed and compared the playground activity of male and female neurotypical and ASD children (*n* = 73; mean age, 7 years). They highlighted that when observing social interactions from a distance, girls with ASD behaved like neurotypical girls, i.e. spending a significant amount of time talking and weaving in and out of groups; yet, it was only upon closer inspection of the quality of interaction with peers that social challenges were perceived. By contrast, it was easier to identify social challenges in boys with ASD at a distance. The authors argued that using camouflaging techniques to mask social difficulties makes girls with ASD more vulnerable and less likely than boys to be identified within a school setting.

This is further supported by Rynkiewicz et al. (2016), in which gestures during ADOS-2 assessments were analysed in 10 girls and 16 boys with high-functioning ASD; girls with ASD tended to use gestures more ‘vividly’ (i.e. with more energy) than boys with ASD (a non-significant trend). The authors hypothesised that girls with ASD are effective at camouflaging such diagnostic features, and this may thereby increase the risk of under-diagnosis in autistic girls. While qualitative differences in gesture use by children with ASD are thought-provoking, they must be considered with caution due to small numbers and lack of a significant group effect.

A paper employing in-depth interviews with females with an Asperger’s diagnosis reported ‘social echolalia’, the act of mimicking socially skilled peers, as a factor that might contribute to the mis- and missed diagnosis of ASD females (Beteta 2008). Another paper using in-depth interviews, this time with parents, adolescent autistic daughters, and siblings, suggested that ASD females are less likely to be identified until the social demands they experience exceed their compensatory strategies (Watson 2014).

During interviews, parents also described their daughters masking their autistic behaviours, adjusting their behaviours to fit in with their peers (Cook et al. 2018). Parents suggested ASD girls were less likely to be identified as autistic by their teachers, due to masking of differences. One parent stated ‘At that stage she was masking and covering up quite well, and although we were aware that there was something not quite right, every time I raised with her teachers at the primary school, … they just said ‘she’s fine’ you know, ‘you’re expecting too much, she’s fine’ (p. 310). Mothers interviewed in one further paper, by Cridland et al. (2014), reported negative consequences from their daughters imitating social behaviours during assessment, and clinicians therefore being unable to identify their autistic behaviours.

### Parental Concerns

Five papers mentioned parental concerns as barriers to diagnosis for females (Duvekot et al. 2017; Little et al. 2017; Navot et al. 2017; Ramsey et al. 2018; Watson 2014). Concerns related to five main themes, i.e. RRBIs, emotional/behavioural problems, social interaction, labelling ASD (i.e. parents considering the possibility of ASD as explaining their daughter’s symptoms), and additional diagnoses.

Ramsey et al. (2018) examined the issues flagged by parents of toddlers at higher likelihood of ASD, who were screened for ASD, and they reported two main effects for gender, in terms of RRBIs and labelling ASD. For RRBIs, parents of boys were found to express concern 1.74 times more than parents of girls, regardless of subsequent diagnosis of the child. The finding for labelling ASD did not reach significance, but this may have been due to small numbers, as it was expressed by the parents of four girls and 22 boys, highlighting that parents of boys were 2.43 times more likely than parents of girls to label ASD itself as a concern for their toddlers who screened positive on ASD questionnaires (again regardless of subsequent diagnosis). When looking at all concerns as a whole, the study found that overall parents expressed one or more concerns about ASD 1.46 times more often for boys than for girls.

Duvekot et al. (2017) also found evidence of differences in terms of parental concerns regarding RRBI type symptoms. Parental reports of RRBI symptoms were significantly less predictive of an ASD diagnosis for females than for males, indicating that even with high scores in this domain, there was less of a link to diagnosis for females. The study also found that one area of parental concern that seemed important for females was emotional and behavioural problems, with females being more likely to receive a diagnosis if levels of these two problem areas were high. Little et al.’s (2017) results indicated that parents of boys with ASD were more likely to express concerns about social interaction prior to diagnosis than parents of girls with ASD.

Two studies dealt exclusively with the aspect of labelling ASD itself for females and recognising symptoms (Navot et al. 2017; Watson 2014). Using semi-structured interviews, Watson (2014) found that parents perceived ASD to be a ‘boy’s disorder’. This perception led to delays in picking up on ASD symptoms. Similarly, Navot et al. (2017) in their study of 11 mother-daughter dyads found that parents expressed concern that they failed to recognise symptoms of ASD in their daughters, for example; ‘I didn’t listen to her. She would just say how she hated school and how she hated visiting my parents and how loud everything is .... I forced her to do all those things. I arranged play dates for her and forced her to go and of course it just made things worse. It was a total failure. And then I got so angry. I was actually furious with her for years .... Although things are very different today, I can’t just push the delete button and pretend that it didn’t happen. We both know I didn’t listen’ (p. 16).

### Others’ Perceptions

Three qualitative studies identified the perceptions of others to be a barrier to female ASD diagnosis, based on interviews conducted with adolescent girls with ASD and their mothers (Cook et al. 2018; Cridland et al. 2014; Navot et al. 2017). The difficulty of having a daughter with a disorder primarily associated with boys was described, for example ‘Most people, once I said, she’s got Asperger’s, would look at me like they didn’t believe me’; ‘We live in a small town. Most people don’t know anyone that has autism, and if they do it’s probably going to be a boy, because autism is sort of a boy kind of issue. And my daughter was so sweet, and people would just say, ‘What’s your problem? Why are you labelling her?’ (Navot et al. 2017, pg. 14). Parents found their concerns were often met with scepticism, however, some adolescent girls with ASD also highlighted positive aspects of ASD being considered a male disorder, for example ‘being surrounded by boys’, because adolescent boys were easier to get along with than adolescent girls (Cridland et al. 2014).

### Lack of Information/Resources

One study indicated that a lack of information was a barrier for the diagnosis of females (Navot et al. 2017). In a qualitative study examining 11 mother-daughter dyads, Navot et al. (2017) found that parents expressed concerns over the lack of information that existed in terms of female ASD, for example ‘I had a hard time finding information that would help me because she was a girl. Everything I read was so much about boys. It was so frustrating and irrelevant. There was just nothing there that could help me figure her out’ (p. 14). Lack of information was seen as detrimental to the diagnostic process, as without relevant and tailored information, parents may not be so inclined to seek diagnosis for their daughters.

Finally, one paper described financial resources as a barrier to ASD diagnosis in females; in focus groups, parents expressed concerns about being able to cover services for their adolescent daughters with ASD (Mademtzi et al. 2018).

### Clinician Bias

Clinician bias was identified by six studies as a barrier to females obtaining an ASD diagnosis (Beteta 2008; Cridland et al. 2014; Giarelli et al. 2010; Mademtzi et al. 2018; Navot et al. 2017; Watson 2014). A large, population-based study, which used case definition to examine the prevalence of ASD (Giarelli et al. 2010), found that boys were more likely than girls to have a diagnosis of ASD even when both sexes had documented ASD symptoms in educational and clinical records. They concluded that this may result from an ‘interpreting bias’, where the observed experiences differ from the expected behaviours dependent on sex bias. They highlighted that clinicians evaluating girls, compared to boys, with a complex developmental profile may be more likely to exclude a classification of ASD if other conditions are present, due to this bias.

Four further papers, using interviews with adolescent girls with ASD and their parents, explored this same theme (Beteta 2008; Cridland et al. 2014; Navot et al. 2017; Watson 2014). Watson (2014), for example, found that parents perceived medical professionals to be hesitant in giving girls a full ASD diagnosis, and instead, they seemed to opt for other diagnoses. Parents also expressed frustration, knowing their daughter was developing atypically, yet not receiving validation from medical professionals about their concerns: ‘We started bringing concerns with our paediatrician really early. A lot of times they were like, oh, she is a late bloomer, but I still felt like there was more going on’; ‘I kept asking to have her evaluated, but with her being a girl, it was even less likely that the paediatrician would refer us. I remember her saying that this is usually a boys’ thing and she is only a little different’ (Navot et al. 2017, pg. 14). Furthermore, findings from Cridland et al. (2014) included reports of healthcare professionals being reluctant to diagnose a female as autistic and a lack of awareness of ASD in females due to a perceived higher incidence of ASD in males.

An additional qualitative paper, this time using focus groups with families, explored the impact of clinician bias. Mademtzi et al. (2018) reported a parent feeling the need to exaggerate their daughter’s impairments to gain a diagnosis; ‘I felt that I needed to make my daughter look more impaired than she actually was, in order to get diagnosis and needed services’. Strict diagnostic criteria also led to delayed diagnosis for females, with one parent saying their daughter was declined diagnosis because ‘she’s two points above the cut-off score’.

## Discussion

We aimed to conduct a mixed-methods systematic review to identify key barriers to obtaining an ASD diagnosis in girls and young women under 21 years. In total, we synthesised 15 quantitative papers, six qualitative, and one mixed-methods study. The two distinct categories/themes describing the data were symptoms and behaviours that are potential barriers to diagnosis and perceived barriers to diagnosis.

For symptoms and behaviours, six themes were identified from the literature, namely behavioural problems, social and communication abilities, language, relationships, additional diagnoses/difficulties and RRBIs. Generally, this review indicated that there are inconsistent findings across papers. This may, in part, be due to the fact that papers focused on different aspects of identified ASD symptoms or behaviours and, also, as there was little consistency in the specific aims or overlap between them. Importantly, this review has highlighted that research to date has included very disparate populations, meaning that direct comparisons between findings are problematic. This is evident in studies recruiting participants from clinical samples (e.g. Petrou et al. 2018), compared to community samples (e.g. Giarelli et al. 2010); also, those limited only to those meeting clinical criteria (e.g. Head et al. 2014) versus those with high-traits of ASD (e.g. Dworzynski et al. 2012; Mandy et al. 2018). It should be noted that clinic samples often have a disproportionately high number of autistic females with lower IQ, and unless this is taken into account, other sex difference may reflect this. Study sample sizes also varied enormously, especially in the number of girls and young women with ASD participating in the study, for example Rynkiewicz et al. (2016) had a sample of 16 compared to 588 in the study by Petrou et al. (2018). The unequal sampling of genders seen in most papers (approximately 80% male samples) may have been implemented in line with the previously accepted 4:1 gender ratio (Rivet and Matson 2011); however, this limits statistical power to find robust gender differences and potentially reduces the likelihood of replication and generalisation.

When looking at behavioural difficulties and language, our results suggest that girls might be being missed within the diagnostic process, and that they require additional problems in verbal ability or behaviour, in order to be noticed and to push them over the diagnostic threshold, a finding which did not occur for boys (Duvekot et al. 2017; Dworzynski et al. 2012; Salomone et al. 2016). This suggests that, for females to be diagnosed using existing criteria, their observable characteristics must be exaggerated to score sufficiently to warrant a diagnosis (Hully and Larmar 2006; Kopp and Gillberg 1992). Therefore, it could be that those girls who have a more subtle presentation of behaviours may be less likely to be referred for a clinical assessment and/or experience longer waiting times. Moreover, if they are assessed, existing diagnostic criteria may not be sufficiently sensitive to identify their needs.

There was mixed evidence in terms of our findings for social communication in females. Mandy et al.’s (2018) findings of sex differences in the onset of social communication difficulties are suggestive of two possible courses, i.e. either females were showing a later onset for autism traits than males or that pre-existing, perhaps more subtle, difficulties were only becoming apparent during adolescence, a time of high social demands. These demands may be greater for females than males (Dunn 2004), which may exceed abilities. Either scenario would have implications in terms of diagnosis, and both may be contributing factors to the later age for initial diagnosis that is often seen for females. On balance, the results suggest that age is a key factor for determining differences in social communication scores between males and females, and this may be pertinent in terms of informing clinical diagnosis across ASD development between childhood and adolescence. Furthermore, a systematic review (Young et al. 2018) has previously outlined specific areas of social communication that are seen more commonly in females than males with ASD. These include ‘desire to interact with others’, ‘better conscience of necessity of social interaction’, ‘passivity perceived as shyness’, ‘tendency to mimic people’, and ‘development of compensatory strategies’ (i.e. camouflaging); however, they did not focus on the transition from childhood to adolescence. Taken as whole, the current review highlights the urgent need for age-specific diagnosis tools that include questions specifically related to those elements of socialisation, and communication deemed difficult for females with ASD.

Only one quantitative paper (Head et al. 2014) examined relationships as a barrier to diagnosis; in this small study, females with ASD showed similar social skills on a friendship and social function questionnaire as neurotypically developing males, demonstrating differences not only from neurotypically developing girls, but also from males with ASD. Again, this difference in terms of relationships might reduce the likelihood of girls being identified for clinical assessment, thereby highlighting a need for greater awareness, as well as gender-specific measures for ASD, specifically in respect of questions on relationships.

In terms of RRBIs, the five papers demonstrated both qualitative and quantitative sex differences. Generally, there was consensus in the findings of the quantitative studies whereby males appeared to present with more RRBIs than females (Duvekot et al. 2017; Tillmann et al. 2018), although one study suggested that RRBIs were more stable over time for females compared to males (Rynkiewicz et al. 2016). A previous systematic review and meta-analysis (of 22 papers) aiming to examine gender differences in the core triad of ASD traits demonstrated similar results that females with ASD show less repetitive and stereotyped behaviour than males (Van Wijngaarden-Cremers et al. 2014). In the current review, the qualitative studies were less consistent, and it is unclear whether female RRBIs are more similar to those of their neurotypical peers or ASD male peers. This area would benefit from more consistent research into potential qualitative differences to aid our understanding of RRBIs, which form part of the diagnostic criteria, to overcome the potential obstacle of only focusing on male-orientated RRBIs. It would also be beneficial to expand the examination of RRBIs beyond looking at only diagnosed females to avoid circularity.

For the perceived barriers section, five core themes were identified, i.e. compensatory behaviours, parental concerns, others’ perceptions, lack of information/resources and clinician bias. However, one overarching concept continuously highlighted as a barrier impacting all levels of identification and diagnosis in females was that of ASD being perceived as a male disorder. It is also noteworthy that in all the papers reviewed, it was primarily parental concerns that were reported, despite this review directly searching for papers that included the perspectives of clinicians, health workers, teachers, and other family members. Therefore, future studies should include a broader range of stakeholders’ perceptions.

Compensatory/camouflaging behaviours were mentioned as a perceived barrier to identification and diagnosis. The fact that girls may be better able than boys to compensate for, or adapt to, aspects of ASD characteristics, described as the ‘camouflage’ hypothesis, has been increasingly documented in the literature (e.g. Hull et al. 2020; Milner et al. 2019). Girls may either intentionally or unconsciously ‘mask’ their social communication difficulties when in social situations (e.g. at school); this has been suggested as a key reason why females may not come to clinical attention and may fail to reach diagnostic thresholds during assessments (Milner et al. 2019). The possible detrimental effect on diagnosis was highlighted in all of the qualitative studies that focused on this topic, where it was identified as being a major barrier for ASD diagnosis in girls specifically; a finding highlighted and strengthened by parental report. These findings suggest that a tool examining the subtle aspects of camouflaging may be beneficial for diagnosis, as well as improved clinical understanding of this phenomenon in girls with ASD. Recently, moves have been made to design such tools, for example the promising work of Hull et al. (2019), who have developed the CAT-Q (Camouflaging Autistic Traits Questionnaire), and Livingston et al. (2020), who have developed a checklist of compensation strategies, which go some way to address these issues. All attempts to design such tools will need to take into account the specific forms of camouflaging, for example mimicking gestures used by peers (Rynkiewicz et al. 2016) or forms of assimilating behavioural traits of peers (Dean et al. 2017).

The findings on parental concerns indicate that parents may experience and express fewer or differing concerns for their autistic daughters than sons. More generally, parents may not even consider ASD as a diagnosis for their daughter(s)—as it has historically been conceived as a ‘male disorder’ (Riley-Hall 2012, p. 37)—and thus, not recognise relevant symptoms or be delayed in doing so. In line with this, parents experienced disbelief and scepticism from others when expressing concerns about their daughters, which was highlighted in three papers (Cook et al. 2018; Cridland et al. 2014; Navot et al. 2017). Additionally, the implication of ASD being perceived as a ‘male disorder’ was noted within the barrier ‘lack of information and resources’, where parents noted they struggled to find specific and relevant information on ASD females. Clinicians also appear to perceive ASD as a male disorder, with parents reporting difficulties from the start of the diagnostic process because of the reluctance of some clinicians to believe females can have ASD. Studies also found that clinicians might be more likely to give females other diagnoses before ASD, potentially contributing directly to delayed diagnosis; it was found that girls who had additional diagnoses were diagnosed with ASD later than those without (Petrou et al. 2018). These current male-centric ideas of autism are detrimental to access to diagnosis and support for autistic females and their families. For any large strides to be made regarding access to services for females, the general public, as well as clinician perceptions of ASD being a male disorder will need to change.

### Limitations

Although a comprehensive search was launched looking at barriers to diagnosis for females with ASD, there were few papers directly addressing this as an issue in terms of specifically extrapolating from straightforward gender comparisons. Comparison of gender-related barriers to diagnosis was often a by-product of the main results rather than the primary focus of included studies. Synthesising evidence of this sort is challenging due to the lack of cohesion in terms of topic focus. Due to the dispersed nature of study designs and findings, quality appraisal tools were adapted as per our requirements. However, piloting procedures ensured the tools used were appropriate. A final limitation was the inclusion of only English language papers thereby reducing cultural variation, which may be a factor in diagnosis.

### Recommendations

There are several clinical implications arising from our review findings. We suggest that clinicians triaging referrals for eligibility for an autism diagnostic assessment consider widening the criteria for females who may seem to have more subtle but longstanding impairments. At assessment, it would be helpful for clinicians to focus on the quality of difficulties, as well as the quantity of presenting symptoms, since females may have a more nuanced clinical presentation. In order to fully understand these nuances, it may also be useful to clarify the trajectory of symptoms, with clients and their significant others (e.g. immediate relatives, partners), such as via a timeline. As part of the assessment, clinicians should ensure that they clarify modifiers for symptoms (i.e. establishing where, when, and with whom these might be more or less prominent). Diagnostic assessment may also benefit from a more systemic focus; for example, finding out about perspectives and narratives about what ASD is, what symptoms are viewed as part of, or distinct from autism (e.g. anxiety, social motivation), and the ways in which this may influence the diagnostic conclusions.

To further enable better clinical decisions, our review strongly suggests that cohesive research on the behavioural profile seen in some females with ASD or ASD traits is required, including an increased understanding of the forms that social communication difficulties and RRBIs might take in this population, as well as an increased awareness of camouflaging behaviours. An important methodological approach in research is to compare community and clinic samples, such as in this review, because this has the advantage of reducing the circularity of only focusing on individuals already diagnosed with ASD using current diagnostic tools that may be gender-biased.

Given that parental perceptions have been studied more extensively, there is also a need for research on perspectives from ASD individuals themselves, clinicians, teachers, and other family members including siblings. One concept that re-occurred throughout our review was that ASD is perceived by stakeholders as a ‘male disorder’. The implications of this are manifold, starting from parents not considering ASD as a potential explanation for their daughters’ difficulties, to lack of information to guide decision-making, and even to clinicians, health professionals, and teachers not recognising ASD as an option for diagnosis for females. All of this points to a vital and urgent need for widespread recognition that ASD is seen in both sexes and all genders, through both research and effective dissemination of knowledge to those in front line positions such as clinicians, teachers, and parents.

## Supplementary Information

ESM 1 (DOCX 44 kb)

ESM 2 (DOCX 18 kb)

## Footnotes

1 Sex/gender is used interchangeably to refer to the inter-related concepts of (biological) sex and (sociocultural) gender.
