# Supplementary material for: Barriers to Autism Spectrum Disorder Diagnosis for Young Women and Girls: a Systematic Review
Source: Rev J Autism Dev Disord. 2020 Oct 29;8(4):454–70. doi: 10.1007/s40489-020-00225-8 (PMC8604819; doi:10.1007/s40489-020-00225-8)
Supplement: Supplementary file 1 — (DOCX 44 kb) [file 40489_2020_225_MOESM1_ESM.docx]

**APPENDIX 2: Adapted Critical Appraisal Skills Programme for Quantitative Papers**

**1. Did the study address a clearly focused issue?**

Yes/No/Can’t Tell

*Hints*:

- A question can be ‘focused’ in terms of
- The population studied
- The risk factors studied
- Is it clear whether the study tried to detect a beneficial or harmful effect
- The outcomes considered

**2. Were the participants recruited in an acceptable way?** (adapted from CASP: “Was the cohort recruited in an acceptable way?”)

Yes/No/Can’t Tell

*Hints*:

- Look for selection bias which might compromise the generalisability of the
  Findings:
  - Was the cohort representative of a defined population?
- Is it acceptable for the study design e.g. clinic vs community setting

***Is it worth continuing?***

**3a. Were the dependent measures accurately assessed to minimise bias?** (adapted from CASP “Was the exposure accurately measured to minimise bias?”)

Yes/No/Can’t Tell

*Hints*:

- Look for measurement bias
  - Did they use subjective or objective measurements
  - Do the measurements truly reflect what you want them to (have they been validated)
  - Were all subjects assessed using the same measures, as relevant to the study design

**3b. Was ASD diagnoses accurately measured to reduce measurement bias?** (adapted from CASP “Was the outcome accurately measured to minimise bias?”

Yes/No/Can’t Tell

*Hints*:

- Look for measurement bias
  - Did they use subjective clinical or/and objective measurements
  - Do the measurements truly reflect what you want them to (have they been validated)
  - Were the measurement methods similar in the different groups

Total 3 = average score of 3a and b

**4a. Have the authors identified all important confounding factors?**

E.g. Age, IQ, SES, language ability, developmental score/level

Yes/No/Can’t Tell

*Hints*:

- List the ones you think might be important, and ones the author missed

**4b. Have they taken account of the confounding factors in the design and/or analysis?**

Yes/No/Can’t Tell

*Hints*:

- Have the confounding factors been included in the analysis?

Total 4 = average score of 4a and b

**5. Do you believe the results?**

*Hints*:

- Consider the size of effect
- Are confidence intervals reported - and if so how precise is the data?
- Can results be due to bias?
  - Very small numbers?
  - Confounding factors not included in the analysis?
  - Poor sampling?
- Are the design and methods of this study sufficiently flawed to make the results unreliable

6**. Are the results generalisable?** (adapted from CASP “Can the results be applied to the local population?”)

*Hints*:

- Sample size
- Type of sample, biased?
  - SES
  - High/low functioning
  - Ethnicity

**7. What are the implications of this study for clinical practice?**

*Hints*:

- Do we think that this has clinical relevance?
- Do they state recommendations for change in clinical practice

**Scoring system**:

Yes: 1 point; Unsure: 0.5 points; No: 0 points
