# Supplementary material for: Barriers to Autism Spectrum Disorder Diagnosis for Young Women and Girls: a Systematic Review
Source: Rev J Autism Dev Disord. 2020 Oct 29;8(4):454–70. doi: 10.1007/s40489-020-00225-8 (PMC8604819; doi:10.1007/s40489-020-00225-8)
Supplement: Supplementary file 2 — (DOCX 18 kb) [file 40489_2020_225_MOESM2_ESM.docx]

# APPENDIX 1: Full database search strategies

| ***Database*** | *Through* | *Database info* | *Date of search* |
| --- | --- | --- | --- |
| **PsycInfo** | OvidSP | PsycINFO 1806 to October Week 2 2018 | 27/10/18 |
| **EMBASE** | OvidSP | Embase 1974 to October Week 2 2018 | 28/10/18 |
| **Medline** | OvidSP | Ovid MEDLINE(R) 1946 to October Week 2 2018 | 27/10/18 |
| **CINAHL** | EBSCOhost | - | 27/10/18 |

# *Overview of search strategy adapted across databases*

These search strategies reflect terms used to identify studies for this review: (Autism Spectrum Disorders AND barriers AND diagnosis AND gender AND children/young people; For objective 1: AND symptoms/behaviours; for objective 2: AND perception AND health worker OR teacher OR parent/family.

| **TEXT WORDS; included in the same way in each database** | **SUBJECT HEADINGS; explored in every database; included where available, adapted as appropriate** | ***PsycInfo*** | ***Embase*** | ***Medline*** | ***CINAHL*** |
| --- | --- | --- | --- | --- | --- |
| **Autism Diagnosis based terms** | | | | | |
| Autis* or Asperger* |  | 🗸 | 🗸 | 🗸 | 🗸 |
| Identif* or diagnos* or screen* or detect* |  | 🗸 | 🗸 | 🗸 | 🗸 |
|  | exp autism assessment/ or exp autism/ or Autism Diagnostic Observation Schedule/ or Autism Diagnostic Interview Revised/ | 🗸 | 🗸 | 🗸 | 🗸 |
|  | exp diagnosis/ or exp early diagnosis/ or exp delayed diagnosis/ or psychiatric diagnosis/ | 🗸 | 🗸 | 🗸 | 🗸 |
| **Barriers to ASD diagnosis based terms** | | | | | |
| barrier* or delayed diagnosis or access* or challenge* |  | 🗸 | 🗸 | 🗸 | 🗸 |
| **Gender and Age based terms** | | | | | |
| child*or adolescent or youth or kid or toddler |  | 🗸 | 🗸 | 🗸 | 🗸 |
| female* or gender or daughter* or girl* or sex or female autis* |  | 🗸 | 🗸 | 🗸 | 🗸 |
|  | exp juvenile/ or exp adolescent/ or exp child/ | 🗸 | 🗸 | 🗸 | 🗸 |
|  | exp "gender and sex"/ or exp gender/ or exp gender bias/ | 🗸 | 🗸 | 🗸 | 🗸 |
|  | exp girl/ or exp female/ | 🗸 | 🗸 | 🗸 | 🗸 |
|  | exp sex/ or exp sex diagnosis/ or exp sex difference/ | 🗸 | 🗸 | 🗸 | 🗸 |
| **Objective 1: Symptom and behaviours based terms** | | | | | |
| symptom* or camo?flag* or mask* or mimic* or compensat* |  | 🗸 | 🗸 | 🗸 | 🗸 |
|  | exp symptom assessment/ or exp biological trait/ or exp behavio?r/di [Diagnosis] | 🗸 | 🗸 | 🗸 | 🗸 |
| **Objective 2: Experience and perception based terms** | | | | | |
| understand* or experienc* or percept* or feel* |  | 🗸 | 🗸 | 🗸 | 🗸 |
|  | exp experience/ or exp personal experience/ | 🗸 | 🗸 | 🗸 | 🗸 |
| **Objective 2: Health workers/ parents/family/ teachers** | | | | | |
| mother* or father* or m?m* or dad* or famil* or parent* or sibling* or sister* or brother* or carer* or caregiver* or guardian* |  | 🗸 | 🗸 | 🗸 | 🗸 |
| teacher* or educator* or school* or uni* or college* |  | 🗸 | 🗸 | 🗸 | 🗸 |
| Health worker* or clinician* or nurse* or doctor* or Paediatric* or psychiatr* or psychologist* or medic* or Physician* |  | 🗸 | 🗸 | 🗸 | 🗸 |
|  | exp parent/ or exp caregiver/ or exp family/ or exp mother/ or exp father/ | 🗸 | 🗸 | 🗸 | 🗸 |
|  | exp health care personnel/ or exp pediatric nurse/ or exp nurse/or exp psychologist/ or exp caregiver/ | 🗸 | 🗸 | 🗸 | 🗸 |
|  | exp teacher/ or exp school teacher/ | 🗸 | 🗸 | 🗸 | 🗸 |

# *Search strategy in each database*

## *EMBASE*

1. exp autism assessment/ or exp autism/ or Autism Diagnostic Observation Schedule/ or Autism Diagnostic Interview Revised/

2. autis*.mp. [mp=title, abstract, heading word, drug trade name, original title, device manufacturer, drug manufacturer, device trade name, keyword, floating subheading word]

3. Asperger*.mp. [mp=title, abstract, heading word, drug trade name, original title, device manufacturer, drug manufacturer, device trade name, keyword, floating subheading word]

4. exp diagnosis/ or exp early diagnosis/ or exp delayed diagnosis/ or psychiatric diagnosis/

5. Identif*.mp. [mp=title, abstract, heading word, drug trade name, original title, device manufacturer, drug manufacturer, device trade name, keyword, floating subheading word]

6. diagnos*.mp. [mp=title, abstract, heading word, drug trade name, original title, device manufacturer, drug manufacturer, device trade name, keyword, floating subheading word]

7. screen*.mp. [mp=title, abstract, heading word, drug trade name, original title, device manufacturer, drug manufacturer, device trade name, keyword, floating subheading word]

8. detect*.mp. [mp=title, abstract, heading word, drug trade name, original title, device manufacturer, drug manufacturer, device trade name, keyword, floating subheading word]

9. exp juvenile/

10. exp adolescent/

11. exp child/

12. exp "gender and sex"/ or exp gender/ or exp gender bias/

13. exp girl/

14. exp female/

15. "wom*n".m_titl.

16. exp sex/ or exp sex diagnosis/ or exp sex difference/

17. female*.mp. [mp=title, abstract, heading word, drug trade name, original title, device manufacturer, drug manufacturer, device trade name, keyword, floating subheading word]

18. gender.mp. [mp=title, abstract, heading word, drug trade name, original title, device manufacturer, drug manufacturer, device trade name, keyword, floating subheading word]

19. Daughter*.mp. [mp=title, abstract, heading word, drug trade name, original title, device manufacturer, drug manufacturer, device trade name, keyword, floating subheading word]

20. girl*.mp. [mp=title, abstract, heading word, drug trade name, original title, device manufacturer, drug manufacturer, device trade name, keyword, floating subheading word]

21. sex.mp. [mp=title, abstract, heading word, drug trade name, original title, device manufacturer, drug manufacturer, device trade name, keyword, floating subheading word]

22. female autis* phenotype*.mp. [mp=title, abstract, heading word, drug trade name, original title, device manufacturer, drug manufacturer, device trade name, keyword, floating subheading word]

23. child*.mp. [mp=title, abstract, heading word, drug trade name, original title, device manufacturer, drug manufacturer, device trade name, keyword, floating subheading word]

24. adolescent.mp. [mp=title, abstract, heading word, drug trade name, original title, device manufacturer, drug manufacturer, device trade name, keyword, floating subheading word]

25. youth.mp. [mp=title, abstract, heading word, drug trade name, original title, device manufacturer, drug manufacturer, device trade name, keyword, floating subheading word]

26. kid.mp. [mp=title, abstract, heading word, drug trade name, original title, device manufacturer, drug manufacturer, device trade name, keyword, floating subheading word]

27. Toddler*.mp. [mp=title, abstract, heading word, drug trade name, original title, device manufacturer, drug manufacturer, device trade name, keyword, floating subheading word]

28. exp symptom assessment/

29. exp biological trait/

30. exp behavior/di [Diagnosis]

31. symptom*.mp. [mp=title, abstract, heading word, drug trade name, original title, device manufacturer, drug manufacturer, device trade name, keyword, floating subheading word]

32. camo?flag*.mp. [mp=title, abstract, heading word, drug trade name, original title, device manufacturer, drug manufacturer, device trade name, keyword, floating subheading word]

33. mask*.mp. [mp=title, abstract, heading word, drug trade name, original title, device manufacturer, drug manufacturer, device trade name, keyword, floating subheading word]

34. mimic*.mp. [mp=title, abstract, heading word, drug trade name, original title, device manufacturer, drug manufacturer, device trade name, keyword, floating subheading word]

35. compensat*.mp. [mp=title, abstract, heading word, drug trade name, original title, device manufacturer, drug manufacturer, device trade name, keyword, floating subheading word]

36. 1 or 2 or 3

37. 4 or 5 or 6 or 7 or 8

38. 9 or 10 or 11 or 23 or 24 or 25 or 26 or 27

39. 12 or 13 or 14 or 15 or 16 or 17 or 18 or 19 or 20 or 21 or 22

40. barrier*.mp. [mp=title, abstract, heading word, drug trade name, original title, device manufacturer, drug manufacturer, device trade name, keyword, floating subheading word]

41. delayed diagnosis.mp. [mp=title, abstract, heading word, drug trade name, original title, device manufacturer, drug manufacturer, device trade name, keyword, floating subheading word]

42. access*.mp. [mp=title, abstract, heading word, drug trade name, original title, device manufacturer, drug manufacturer, device trade name, keyword, floating subheading word]

43. challenge*.mp. [mp=title, abstract, heading word, drug trade name, original title, device manufacturer, drug manufacturer, device trade name, keyword, floating subheading word]

44. health service* access*.mp. [mp=title, abstract, heading word, drug trade name, original title, device manufacturer, drug manufacturer, device trade name, keyword, floating subheading word]

45. health system* access*.mp. [mp=title, abstract, heading word, drug trade name, original title, device manufacturer, drug manufacturer, device trade name, keyword, floating subheading word]

46. 40 or 41 or 42 or 43 or 44 or 45

47. 36 and 37 and 38 and 39 and 46

48. 28 or 29 or 30 or 31 or 32 or 33 or 34 or 35

49. 47 and 48 [Objective 1]

50. exp experience/ or exp personal experience/

51. understand*.mp. [mp=title, abstract, heading word, drug trade name, original title, device manufacturer, drug manufacturer, device trade name, keyword, floating subheading word]

52. experienc*.mp. [mp=title, abstract, heading word, drug trade name, original title, device manufacturer, drug manufacturer, device trade name, keyword, floating subheading word]

53. percept*.mp. [mp=title, abstract, heading word, drug trade name, original title, device manufacturer, drug manufacturer, device trade name, keyword, floating subheading word]

54. feel*.mp. [mp=title, abstract, heading word, drug trade name, original title, device manufacturer, drug manufacturer, device trade name, keyword, floating subheading word]

55. 50 or 51 or 52 or 53 or 54

56. exp health care personnel/

57. exp pediatric nurse/ or exp nurse/

58. exp psychologist/

59. exp caregiver/

60. Health worker*.mp. [mp=title, abstract, heading word, drug trade name, original title, device manufacturer, drug manufacturer, device trade name, keyword, floating subheading word]

61. clinician*.mp. [mp=title, abstract, heading word, drug trade name, original title, device manufacturer, drug manufacturer, device trade name, keyword, floating subheading word]

62. nurse*.mp. [mp=title, abstract, heading word, drug trade name, original title, device manufacturer, drug manufacturer, device trade name, keyword, floating subheading word]

63. doctor*.mp. [mp=title, abstract, heading word, drug trade name, original title, device manufacturer, drug manufacturer, device trade name, keyword, floating subheading word]

64. Paediatric*.mp. [mp=title, abstract, heading word, drug trade name, original title, device manufacturer, drug manufacturer, device trade name, keyword, floating subheading word]

65. psychiatr*.mp. [mp=title, abstract, heading word, drug trade name, original title, device manufacturer, drug manufacturer, device trade name, keyword, floating subheading word]

66. psychologist*.mp. [mp=title, abstract, heading word, drug trade name, original title, device manufacturer, drug manufacturer, device trade name, keyword, floating subheading word]

67. medic*.mp. [mp=title, abstract, heading word, drug trade name, original title, device manufacturer, drug manufacturer, device trade name, keyword, floating subheading word]

68. caregiver*.mp. [mp=title, abstract, heading word, drug trade name, original title, device manufacturer, drug manufacturer, device trade name, keyword, floating subheading word]

69. Physician*.mp. [mp=title, abstract, heading word, drug trade name, original title, device manufacturer, drug manufacturer, device trade name, keyword, floating subheading word]

70. 56 or 57 or 58 or 59 or 60 or 61 or 62 or 63 or 64 or 65 or 66 or 67 or 68 or 69

71. exp teacher/ or exp school teacher/

72. teacher*.mp. [mp=title, abstract, heading word, drug trade name, original title, device manufacturer, drug manufacturer, device trade name, keyword, floating subheading word]

73. educator*.mp. [mp=title, abstract, heading word, drug trade name, original title, device manufacturer, drug manufacturer, device trade name, keyword, floating subheading word]

74. school*.mp. [mp=title, abstract, heading word, drug trade name, original title, device manufacturer, drug manufacturer, device trade name, keyword, floating subheading word]

75. student.mp. [mp=title, abstract, heading word, drug trade name, original title, device manufacturer, drug manufacturer, device trade name, keyword, floating subheading word]

76. exp student/

77. 71 or 72 or 73 or 74 or 75 or 76

78. exp parent/

79. exp caregiver/

80. exp family relation/ or exp human relation/ or child parent relation/ or exp father child relation/ or exp sibling relation/

81. exp family/

82. exp mother/

83. exp father/

84. mother*.mp. [mp=title, abstract, heading word, drug trade name, original title, device manufacturer, drug manufacturer, device trade name, keyword, floating subheading word]

85. father*.mp. [mp=title, abstract, heading word, drug trade name, original title, device manufacturer, drug manufacturer, device trade name, keyword, floating subheading word]

86. m?m*.mp. [mp=title, abstract, heading word, drug trade name, original title, device manufacturer, drug manufacturer, device trade name, keyword, floating subheading word]

87. dad*.mp. [mp=title, abstract, heading word, drug trade name, original title, device manufacturer, drug manufacturer, device trade name, keyword, floating subheading word]

88. famil*.mp. [mp=title, abstract, heading word, drug trade name, original title, device manufacturer, drug manufacturer, device trade name, keyword, floating subheading word]

89. parent*.mp. [mp=title, abstract, heading word, drug trade name, original title, device manufacturer, drug manufacturer, device trade name, keyword, floating subheading word]

90. sibling*.mp. [mp=title, abstract, heading word, drug trade name, original title, device manufacturer, drug manufacturer, device trade name, keyword, floating subheading word]

91. sister*.mp. [mp=title, abstract, heading word, drug trade name, original title, device manufacturer, drug manufacturer, device trade name, keyword, floating subheading word]

92. brother*.mp. [mp=title, abstract, heading word, drug trade name, original title, device manufacturer, drug manufacturer, device trade name, keyword, floating subheading word]

93. carer*.mp. [mp=title, abstract, heading word, drug trade name, original title, device manufacturer, drug manufacturer, device trade name, keyword, floating subheading word]

94. guardian*.mp. [mp=title, abstract, heading word, drug trade name, original title, device manufacturer, drug manufacturer, device trade name, keyword, floating subheading word]

95. 78 or 79 or 80 or 81 or 82 or 83 or 84 or 85 or 86 or 87 or 88 or 89 or 90 or 91 or 92 or 93 or 94

96. 47 and 55 and 70

97. 47 and 55 and 77

98. 47 and 55 and 95

99. 96 or 97 or 98 [Objective 2]

***PsychInfo***

1. exp autism assessment/ or exp autism/ or Autism Diagnostic Observation Schedule/ or Autism Diagnostic Interview Revised.mp. [mp=title, abstract, heading word, table of contents, key concepts, original title, tests & measures]

2. autis*.mp.

3. Asperger*.mp.

4. exp diagnosis/ or exp early diagnosis/ or exp delayed diagnosis/ or psychiatric diagnosis

5. identif*.mp.

6. diagnos*.mp.

7. screen*.mp.

8. detect*.mp.

9. juvenile.mp.

10. adolescent.mp.

11. child.mp.

12. (gender and sex).mp. [mp=title, abstract, heading word, table of contents, key concepts, original title, tests & measures]

13. gender.mp.

14. gender bias.mp.

15. exp girl/

16. female.mp.

17. woman.mp. [mp=title, abstract, heading word, table of contents, key concepts, original title, tests & measures]

18. women.mp. [mp=title, abstract, heading word, table of contents, key concepts, original title, tests & measures]

19. exp sex/

20. sex diagnosis.mp.

21. sex difference.mp.

22. female*.mp.

23. daughter*.mp.

24. girl*.mp.

25. sex.mp.

26. female autism* phenotype*.mp.

27. female autistic phenotype.mp.

28. child*.mp.

29. adolescent.mp.

30. youth.mp.

31. kid.mp.

32. toddler*.mp.

33. symptom assessment.mp.

34. biological trait.mp.

35. behaviour.mp.

36. behavior.mp. [mp=title, abstract, heading word, table of contents, key concepts, original title, tests & measures]

37. symptom*.mp.

38. camouflage*.mp.

39. camoflage.mp.

40. mask*.mp.

41. mimic*.mp.

42. compensation*.mp.

43. compensatory*.mp.

44. 1 or 2 or 3

45. 4 or 5 or 6 or 7 or 8

46. 9 or 10 or 11 or 28 or 29 or 30 or 31 or 32

47. 12 or 13 or 14 or 15 or 16 or 17 or 18 or 19 or 20 or 21 or 22 or 23 or 24 or 25 or 26 or 27

48. barrier*.mp.

49. delayed diagnosis.mp.

50. access*.mp.

51. challenge*.mp.

52. health service* access*.mp.

53. health system* access*.mp.

54. 48 or 49 or 50 or 51 or 52 or 53

55. 44 and 45 and 46 and 47 and 54

56. 33 or 34 or 35 or 36 or 37 or 38 or 39 or 40 or 41 or 42 or 43

57. 55 and 56

58. experience.mp.

59. personal experience.mp.

60. understand*.mp.

61. experienc*.mp.

62. percept*.mp.

63. feel*.mp.

64. 58 or 59 or 60 or 61 or 62 or 63

65. health care personnel.mp.

66. pediatric nurse.mp.

67. nurse.mp.

68. psychologist.mp.

69. caregiver.mp.

70. health worker*.mp.

71. clinician*.mp.

72. nurse*.mp.

73. doctor*.mp.

74. pediatric*.mp.

75. psychiatr*.mp.

76. psychologist*.mp.

77. medic*.mp.

78. caregiver*.mp.

79. physician*.mp.

80. 65 or 66 or 67 or 68 or 69 or 70 or 71 or 72 or 73 or 74 or 75 or 76 or 77 or 78 or 79

81. teacher.mp.

82. school teacher.mp.

83. teacher*.mp.

84. educator*.mp.

85. school*.mp.

86. student.mp.

87. 81 or 82 or 83 or 84 or 85 or 86

88. parent.mp.

89. caregiver.mp.

90. exp family relation/ or exp human relation/ or child parent relation/ or exp father child relation/ or exp sibling relation/

91. exp family/

92. mother.mp.

93. father.mp.

94. mother*.mp.

95. father*.mp.

96. mum*.mp.

97. mom*.mp.

98. dad*.mp.

99. famil*.mp.

100. parent*.mp.

101. sibling*.mp.

102. sister*.mp.

103. brother*.mp.

104. carer*.mp.

105. guardian*.mp.

106. 88 or 89 or 90 or 91 or 92 or 93 or 94 or 95 or 96 or 97 or 98 or 99 or 100 or 101 or 102 or 103 or 104 or 105

107. 55 and 64 and 80

108. 55 and 64 and 87

109. 55 and 64 and 106

110. 107 or 108 or 109

***Medline***

1. exp autism assessment/ or exp autism/ or Autism Diagnostic Observation Schedule/ or Autism Diagnostic Interview Revised/

2. autis*.mp.

3. Asperger*.mp.

4. exp diagnosis/ or exp early diagnosis/ or exp delayed diagnosis/ or psychiatric diagnosis/

5. Identif*.mp.

6. diagnos*.mp.

7. screen*.mp.

8. detect*.mp.

9. juvenile.mp.

10. exp adolescent/

11. exp child/

12. exp "gender and sex"/ or exp gender/ or exp gender bias/

13. exp girl/

14. exp female/

15. wom#n.mp.

16. exp sex/ or exp sex diagnosis/ or exp sex difference/

17. female*.mp.

18. gender.mp.

19. Daughter*.mp.

20. girl*.mp.

21. sex.mp.

22. female autis* phenotype*.mp.

23. child*.mp.

24. adolescent.mp.

25. youth.mp.

26. kid.mp.

27. Toddler*.mp.

28. exp symptom assessment/

29. biological trait.mp.

30. exp behavior/di [Diagnosis]

31. symptom*.mp.

32. camo?flag*.mp.

33. mask*.mp.

34. mimic*.mp.

35. compensat*.mp.

36. 1 or 2 or 3

37. 4 or 5 or 6 or 7 or 8

38. 9 or 10 or 11 or 23 or 24 or 25 or 26 or 27

39. 12 or 13 or 14 or 15 or 16 or 17 or 18 or 19 or 20 or 21 or 22

40. barrier*.mp.

41. delayed diagnosis.mp.

42. access*.mp.

43. challenge*.mp.

44. health service* access*.mp.

45. health system* access*.mp.

46. 40 or 41 or 42 or 43 or 44 or 45

47. 36 and 37 and 38 and 39 and 46

48. 28 or 29 or 30 or 31 or 32 or 33 or 34 or 35

49. 47 and 48

50. (experience or personal experience).mp. [mp=ti, ab, hw, tn, ot, dm, mf, dv, kw, fx, dq, nm, kf, px, rx, ui, sy, tc, id, tm]

51. understand*.mp.

52. experienc*.mp.

53. percept*.mp.

54. feel*.mp.

55. 50 or 51 or 52 or 53 or 54

56. health care personnel.mp.

57. exp p?ediatric nurse/ or exp nurse/

58. exp psychologist/

59. exp caregiver/

60. Health worker*.mp. [mp=ti, ab, hw, tn, ot, dm, mf, dv, kw, fx, dq, nm, kf, px, rx, ui, sy, tc, id, tm]

61. clinician*.mp.

62. nurse*.mp. [mp=ti, ab, hw, tn, ot, dm, mf, dv, kw, fx, dq, nm, kf, px, rx, ui, sy, tc, id, tm]

63. doctor*.mp. [mp=ti, ab, hw, tn, ot, dm, mf, dv, kw, fx, dq, nm, kf, px, rx, ui, sy, tc, id, tm]

64. P?ediatric*.mp. [mp=ti, ab, hw, tn, ot, dm, mf, dv, kw, fx, dq, nm, kf, px, rx, ui, sy, tc, id, tm]

65. psychiatr*.mp. [mp=ti, ab, hw, tn, ot, dm, mf, dv, kw, fx, dq, nm, kf, px, rx, ui, sy, tc, id, tm]

66. psychologist*.mp. [mp=ti, ab, hw, tn, ot, dm, mf, dv, kw, fx, dq, nm, kf, px, rx, ui, sy, tc, id, tm]

67. medic*.mp.

68. caregiver*.mp. [mp=ti, ab, hw, tn, ot, dm, mf, dv, kw, fx, dq, nm, kf, px, rx, ui, sy, tc, id, tm]

69. Physician*.mp. [mp=ti, ab, hw, tn, ot, dm, mf, dv, kw, fx, dq, nm, kf, px, rx, ui, sy, tc, id, tm]

70. 56 or 57 or 58 or 59 or 60 or 61 or 62 or 63 or 64 or 65 or 66 or 67 or 68 or 69

71. (teacher or school teacher).mp. [mp=ti, ab, hw, tn, ot, dm, mf, dv, kw, fx, dq, nm, kf, px, rx, ui, sy, tc, id, tm]

72. teacher*.mp.

73. educator*.mp.

74. school*.mp.

75. student.mp.

76. exp student/

77. 71 or 72 or 73 or 74 or 75 or 76

78. exp parent/

79. exp caregiver/

80. exp family relation/ or exp human relation/ or child parent relation/ or exp father child relation/ or exp sibling relation/

81. exp family/

82. exp mother/

83. exp father/

84. mother*.mp. [mp=ti, ab, hw, tn, ot, dm, mf, dv, kw, fx, dq, nm, kf, px, rx, ui, sy, tc, id, tm]

85. father*.mp. [mp=ti, ab, hw, tn, ot, dm, mf, dv, kw, fx, dq, nm, kf, px, rx, ui, sy, tc, id, tm]

86. m?m*.mp. [mp=ti, ab, hw, tn, ot, dm, mf, dv, kw, fx, dq, nm, kf, px, rx, ui, sy, tc, id, tm]

87. dad*.mp. [mp=ti, ab, hw, tn, ot, dm, mf, dv, kw, fx, dq, nm, kf, px, rx, ui, sy, tc, id, tm]

88. famil*.mp. [mp=ti, ab, hw, tn, ot, dm, mf, dv, kw, fx, dq, nm, kf, px, rx, ui, sy, tc, id, tm

89. parent*.mp. [mp=ti, ab, hw, tn, ot, dm, mf, dv, kw, fx, dq, nm, kf, px, rx, ui, sy, tc, id, tm]

90. sibling*.mp. [mp=ti, ab, hw, tn, ot, dm, mf, dv, kw, fx, dq, nm, kf, px, rx, ui, sy, tc, id, tm]

91. sister*.mp. [mp=ti, ab, hw, tn, ot, dm, mf, dv, kw, fx, dq, nm, kf, px, rx, ui, sy, tc, id, tm]

92. brother*.mp. [mp=ti, ab, hw, tn, ot, dm, mf, dv, kw, fx, dq, nm, kf, px, rx, ui, sy, tc, id, tm]

93. carer*.mp. [mp=ti, ab, hw, tn, ot, dm, mf, dv, kw, fx, dq, nm, kf, px, rx, ui, sy, tc, id, tm]

94. guardian*.mp. [mp=ti, ab, hw, tn, ot, dm, mf, dv, kw, fx, dq, nm, kf, px, rx, ui, sy, tc, id, tm]

95. 78 or 79 or 80 or 81 or 82 or 83 or 84 or 85 or 86 or 87 or 88 or 89 or 90 or 91 or 92 or 93 or 94

96. 47 and 55 and 70

97. 47 and 55 and 77

98. 47 and 55 and 95

99. 96 or 97 or 98

***CINAHL***

1. autis*
2. exp autism assessment/ or exp autism/ or Autism Diagnostic Observation Schedule/ or Autism Diagnostic Interview Revised/
3. asperger*
4. exp diagnosis/ or exp early diagnosis/ or exp delayed diagnosis/ or psychiatric diagnosis/
5. Identif*
6. diagnos*
7. screen*
8. detect*
9. juvenile
10. adolescen*
11. child
12. sex
13. girl*
14. wom#n
15. female*
16. gender
17. daughter*
18. female autis* phenotype*
19. child*
20. youth
21. kids
22. toddler*
23. symptom assessment
24. biological traits
25. behavior
26. diagnosis
27. symptom*
28. camo#flag*
29. mask*
30. mimic*
31. compensat*
32. S1 OR S2 OR S3
33. S4 OR S5 OR S6 OR S7 OR S8
34. S9 OR S10 OR S11 OR S19 OR S20 OR S21 OR S22
35. S12 OR S13 OR S14 OR S15 OR S16 OR S17 OR S18
36. S23 OR S24 OR S25 OR S26 OR S27 OR S28 OR S29 OR S30 OR S31
37. barrier*
38. delayed diagnosis or late diagnosis
39. delayed diagnosis or late diagnosis
40. access*
41. challenge*
42. health service* access*
43. health system* access*
44. S37 OR S38 OR S39 OR S40 OR S41 OR S42 OR S43
45. S32 AND S33 AND S34 AND S35 AND S36 AND S44
46. experience or personal experience
47. understand*
48. experienc*
49. percept*
50. feel*
51. S46 OR S47 OR S48 OR S49 OR S50
52. health care personnel
53. p#ediatric nurse
54. nurse
55. psychologist
56. caregiver
57. health workers
58. clinician
59. doctors
60. p#ediatric
61. psychiatr*
62. psychologist*
63. medic*
64. caregiver*
65. physician*
66. S52 OR S53 OR S54 OR S55 OR S56 OR S57 OR S58 OR S59 OR S60 OR S61 OR S62 OR S63 OR S64 OR S65
67. teacher*
68. educator*
69. school*
70. students
71. S67 OR S68 OR S69 OR S70
72. parents
73. caregiver
74. family relation*
75. child parent relation*
76. sibling relation*
77. family
78. mother*
79. father*
80. m?m
81. dad*
82. famil*
83. parent*
84. sibling*
85. sister*
86. brother*
87. carer*
88. guardian*
89. S72 OR S73 OR S74 OR S75 OR S76 OR S77 OR S78 OR S79 OR S80 OR S81 OR S82 OR S83 OR S84 OR S85 OR S86 OR S87 OR S88
90. S32 AND S33 AND S34 AND S35 AND S44
91. S51 AND S66 AND S90
92. S51 AND S71 AND S90
93. S51 AND S89 AND S90
94. S91 OR S92 OR S93
95. S91 OR S92 OR S93
